# Simple and Rapid HPLC Separation and Quantification of Flavonoid, Flavonolignans, and 2,3-Dehydroflavonolignans in Silymarin

**DOI:** 10.3390/foods9020116

**Published:** 2020-01-21

**Authors:** Lucie Petrásková, Kristýna Káňová, David Biedermann, Vladimír Křen, Kateřina Valentová

**Affiliations:** 1 Institute of Microbiology of the Czech Academy of Sciences, Vídeňská 1083, CZ 142 20 Prague, Czech Republic; petraskova@biomed.cas.cz (L.P.); astriik@gmail.com (K.K.); kren@biomed.cas.cz (V.K.); 2 Department of Biochemistry and Microbiology, University of Chemistry and Technology Prague, Technická 5, CZ 166 28 Prague, Czech Republic

**Keywords:** silymarin, milk thistle, flavonolignans, HPLC-MS separation, quantification, diastereoisomers, enantiomers

## Abstract

Herbal preparations from *Silybum marianum* have been used since the fourth century BC in liver disease treatment and against numerous other pathologies. Consumption of silymarin containing drugs and food supplements continues to increase. Precise, fast, reliable, and complex determination of all components of silymarin preparations is paramount for assessing its pharmacological quality. We present here simple and fast HPLC-DAD and LC-MS analytical methods for the determination and quantification of all known silymarin components, including 2,3-dehydroflavonolignans that has not been achieved so far. The first method, using a common C18 column, allows baseline separation of previously inseparable silychristin A, B, isosilychristin, and silydianin. Moreover, this method allowed detection of three so far unknown silymarin components. In addition, the first analytical separation of enantiomers of 2,3-dehydrosilybin was achieved using a Lux 3μ Cellulose-4 chiral column, providing even more accurate description of silymarin composition. 2,3-Dehydroflavonolignans were isolated for the first time from silymarin using preparative chromatography on C18 and ASAHIPAK columns, and 2,3-dehydrosilychristin and 2,3-dehydrosilybin were for the first time conclusively confirmed by HPLC, MS, and NMR to be silymarin components. Using the optimized analytical methods, six various silymarin preparations were analyzed showing substantial differences in the composition.

## 1. Introduction

**Figure 1:**
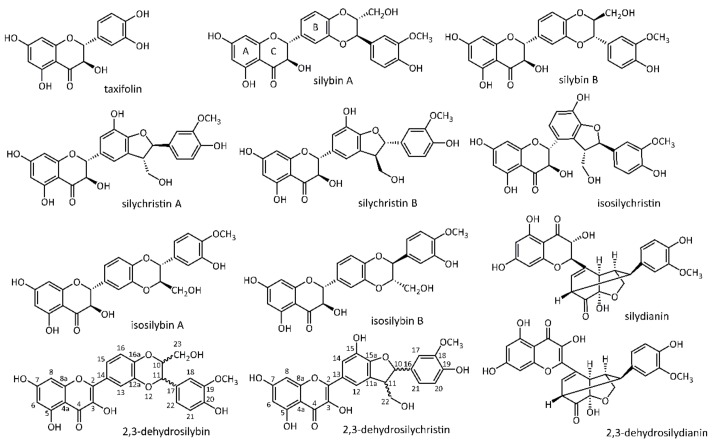
Structures of silymarin components.

*Silybum marianum* (L.) Gaertn (Asteraceae)—milk thistle—is an annual or biennial herb well known for its therapeutic effects since ancient times. The flowers, roots [1], and mainly the fruits (achenes) contain a rather unique type of polyphenols, the flavonolignans such as isosilychristin, silychristins A and B, silydianin, silybins A and B, isosilybins A and B (Figure 1). The complex extract containing them and known as silymarin (SM) [2,3,4] is obtained from milk thistle fruits and contains besides the flavonolignans also their biogenetic precursor the flavanonol taxifolin [5]. The corresponding 2,3-dehydroderivatives of flavonolignans such as 2,3-dehydrosilybin and 2,3-dehydrosilychristin (Figure 1) are tentative silymarin components as well although they used to be considered possible artifacts or oxidative products arising from processing and storage [6]. Moreover, dry silymarin consists of up to 30–40% of polymeric phenolic fraction of unknown composition, contained already in the intact plant material but formed also during the processing and storage [7]. Therefore, silymarin is a very complex mixture of structurally related compounds that is very difficult to analyze in a single run and at reasonable time.

Thanks to their easy isolation from silymarin [8], silybins A and B or their diastereomeric mixture denoted as silybin or silibinin are usually considered to be the main components of silymarin and the biological activities of the whole complex are mostly ascribed to them. Nevertheless, there is evidence that the individual components contained in silymarin may be selectively responsible for various bioactivities. For example, silybin B and taxifolin influence estrogen responsive plasmid construct in breast cancer cells in vitro [9]. Furthermore, isosilybin B suppresses the cell growth of prostate carcinoma cells in culture [10] and inhibits cytochrome P450 3A4 in human liver microsomes [11]. It was also discovered that pure silybins A and B have entirely different metabolic profiles in rat plasma [12] and 2,3-dehydrosilybin A was the most active pro-longevity compound in *Caenorhabditis elegans* [13]. A different ability of 2,3-dehydrosilybin, silychristin, isosilybin B, and silydianin to reduce UVA-stimulated cellular damage to primary human fibroblasts was also observed [14]. Skin intake of silybin, 2,3-dehydrosilybin, and isosilybin was greater than that of taxifolin, silychristin, and silydianin [15]. 2,3-Dehydrosilybin showed the highest UVA protection factor [16] and inhibited basal cell carcinoma growth both in vitro and in a mice allograft model to a greater extent than silybin [17]. Thus, the minority compounds in silymarin should not be neglected since they are highly biologically active as isolated species or they act as a complex in synergy with other silymarin constituents.

However, most of the silymarin manufacturers characterize their products by the percentage amounts of silybins, silychristins, and isosilybins only, leaving out the information of the other (minor) compounds, not to mention the polymeric fraction. Moreover, manufacturers’ claims of substance content often do not correlate with the reality. The large discrepancies between the declared and experimentally observed silymarin composition (in the range of 35–125%) were recently published [18]. Since this is a plant material, it can be assumed that the content of flavonolignans in silymarin depends, e.g., on the growing location, agricultural techniques, climate, storage, and extraction methods. All these variables influence the ratio of the individual compounds in silymarin and thus each batch (even from the same manufacturer) of silymarin is rather unique [7,18,19,20]. A precise analytical determination of silymarin components is therefore essential prior to any biological study to keep reproducibility and validity of the results.

The universal analytical method for the identification of silymarin components is the high-performance liquid chromatography with the spectrometric detection (HPLC-UV). The most commonly used stationary phase is C18 and various mobile phases can be used (typically combination of acetonitrile and/or methanol with water in acidic conditions) either in isocratic or in a gradient mode. All these methods usually yielded good separation of taxifolin, silychristin A, silybins A and B, and isosilychristins A and B in the time range from 7 to 100 min. However, a fundamental issue is the separation of silychristin B and silydianin, because these compounds co-elute or separate only partially [7,19]. Moreover, these separation methods completely disregarded 2,3-dehydroflavonolignans such as 2,3-dehydrosilybin and 2,3-dehydrosilychristin being present in silymarin although in small quantities. In addition, 2,3-dehydroderivatives of silymarin flavonolignans occur naturally in the form of enantiomers [8]. To the best of our knowledge, HPLC separation of all these individual enantiomers has not been published yet.

Here, we report on simple HPLC and LC-MS analytical methods for the rapid determination and quantification of all up to now known silymarin components including 2,3-dehydroflavonolignans. Furthermore, we describe a novel method for the separation of 2,3-dehydrosilybin enantiomers A and B.

## 2. Materials and Methods 

### 2.1. Material

Silymarin was obtained from the following suppliers: Sigma-Aldrich (SM 1; St. Louis, MO, USA, batch No. BCBJ0393V), Liaoning Senrong Pharmaceuticals (SM 2; Panjin, China, batch No. 120501), INDENA (SM 3; Settala, Italy, batch No. 32621/M5), Panjin Huacheng Pharmaceutical Company (SM 4; with an additive for better solubility in water, Panjin, China, batch No. E5S66), Takeda (SM 5; Konstanz, Germany, Flavobion^®^ coated tablets, batch No. 383036), Panjin Huacheng Pharmaceutical Company (SM 6; supplied in August 2019 without batch No., Panjin, China). Flavobion^®^ coated tablets (25 tablets, 11.360 g) were powdered and subjected to Soxhlet extraction with acetone (450 mL) for 2 h, the extract was evaporated in vacuo to yield the sample SM 5 (126 mg of dry extract). The extract was stored at −80 °C. Standards of silybin A, silybin B, 2,3-*cis-*silybin A, 2,3-*cis-*silybin B, 10,11-*cis*-silybin A, 10,11-*cis*-silybin A, silychristin A, silychristin B, isosilychristin, silydianin, isosilybin A, isosilybin B, silyhermin, 2,3-dehydrosilybin A, 2,3-dehydrosilybin B, 2,3-dehydrosilychristin A, 2,3-dehydrosilychristin B, 2,3-dehydroisosilybin, and 2,3-dehydrosilydianin were prepared and fully characterized in the Laboratory of Biotransformation, Institute of Microbiology, Prague, CZ [11,21,22,23,24,25]. Taxifolin was purchased from Amagro (Prague, Czech Republic) and coniferyl alcohol from Sigma-Aldrich (Merck, Kenilworth, NJ, USA). All standard solutions for calibration curves were prepared in dimethyl sulfoxide in volumetric flasks. The silymarin preparations SM 1–SM 6 were also dissolved in dimethyl sulfoxide and their concentrations were 10.3, 6.5, 13.3, 8.3, 15.5, and 23.1 mg/mL, respectively. All substances dissolved in dimethyl sulfoxide were stable during the measurement; no new peaks appeared in repeated measurements even after several weeks. The concentrations of the flavanonol, flavonolignans, and 2,3-dehydroflavonolignans were calculated using seven-point calibration curves.

Silymarin fraction containing concentrated 2,3-dehydroderivatives of flavonolignans was obtained from silymarin (SM 2 preparation) as described previously [23] using Sephadex LH-20 glass column XK 50 (100 × 5 cm, bead size 25–100 μm, GE Healthcare Life Sciences, Pittsburgh, PA, USA) equipped with a thermostatic jacket (23 °C). Isocratic elution with methanol, flow rate 3 mL/min, volume of each fraction was 30 mL, UV detection at 254 nm, run-time 28 h [23]. Briefly, 6 g of “silybin free” silymarin (see Appendix B) was loaded onto the column and eluted with methanol to obtain a fraction enriched in 2,3-dehydroderivatives of flavonolignans (typically 0.8 g).

Acetonitrile, methanol, formic acid, dimethyl sulfoxide (Avantor, Radnor, PA, USA), and deionized water were of LC-MS grade.

### 2.2. Analytical Methods

HPLC and LC-MS analyses were performed on a Shimadzu Prominence LC analytical system consisting of Shimadzu LC-20AD binary HPLC pump, Shimadzu CTO-10AS column oven, Shimadzu SIL-20ACHT cooling autosampler, Shimadzu CBM-20A system controller, and Shimadzu SPD-20MA diode array detector; LC-MS-2020 mass detector with single quadrupole equipped with an electrospray ionization (all Shimadzu, Kyoto, Japan).

#### 2.2.1. Isocratic Method

The original isocratic method for the separation of silymarin components was performed under isocratic conditions on monolithic Chromolith RP-18e (100 × 3 mm) column with monolithic Chromolith RP-18e (5 × 4.6 mm) pre-column (both Merck, Darmstadt, Germany), mobile phase: 2% acetonitrile, 37% methanol, 0.1% formic acid, flow rate 1.1 mL/min, t = 25 °C. The PDA data were acquired in the 200–400 nm range, sampling 40 Hz, time constant 0.025 s, and signals at 285 nm were extracted. The injection volume was 2 μL.

#### 2.2.2. Gradient Method

The quantification was performed on monolithic Chromolith RP-C18 column (100 × 3 mm) equipped with guard cartridge Chromolith RP-18e (5 × 4.6 mm; both Merck, Darmstadt, Germany) under binary gradient elution (mobile phase: A = 5% acetonitrile, 0.1% formic acid; B = 80% methanol, 0.1% formic acid; gradient: 0 min 30% B, 12 min 60% B, 13 min 60% B, 14 min 30% B, 16.5 min stop), flow rate 1.1 mL/min, 14–16 min 1.5 mL/min for faster re-equilibration of the column, t = 25 °C. The PDA data were acquired in the 200–400 nm range, sampling 40 Hz, time constant 0.025 s, and signals at 285 nm were extracted. The injection volume was 2 µL. 

#### 2.2.3. Gradient Method for 2,3-dehydroflavonolignans

2,3-Dehydroflavonolignans from silymarin were analyzed by an LC-MS method on monolithic Chromolith RP-18e (100 × 3 mm) column (Merck, Darmstadt, Germany), Chromolith RP-18e (5 × 4.6 mm) pre-column (Merck, Darmstadt, Germany). Binary gradient elution was performed using mobile phase A = 5% acetonitrile, 0.1% formic acid, B = 80% acetonitrile, 0.1% formic acid, and gradient: 0 min 20% B, 5 min 90% B, 6 min 90% B, 8–10 min 20% B; flow rate 0.4 mL/min, 25 °C, MS detection. The MS parameters were as follows: Negative mode; ESI interface voltage, 4.5 kV; detector voltage, 1.15 kV; nebulizing gas flow 1.5 mL/min; drying gas flow 15 mL/min; heat block temperature 200 °C; DL temperature 250 °C; SCAN mode 450–650 *m*/*z*. Spectra were extracted in the 479.0–479.1 *m*/*z* range ([M-H]^−^ ions of 2,3-dehydroflavonolignans) using LabSolutions software version 5.75 SP2 (Shimadzu, Kyoto, Japan). The injection volume was 1 μL.

#### 2.2.4. Chiral Separation of Enantiomers of 2,3-dehydroflavonolignans

The separation of 2,3-dehydrosilybin A and 2,3-dehydrosilybin B was achieved on Lux 3μ Cellulose-4 (2 × 50 mm, particle size 3 µm) column (Phenomenex, Torrance, CA, USA) equipped with security guard cartridge Lux Cellulose-4 (4 × 2.0 mm, 3 μm, Phenomenex, Torrance, CA, USA). Binary gradient elution: Mobile phase A: 10% acetonitrile, 0.1% formic acid; mobile phase B: 80% acetonitrile, 0.1% formic acid; gradient: 0 min 30% B, 0–12 min 30–50% B, 12–13 min 50–30% B, 13–15 min 30% B to equilibrate the column; flow rate 0.5 mL/min, t = 25 °C. The sample was dissolved in methanol and injection volume was 1 μL.

### 2.3. Validation of the Analytical Chromatographic Method

Relative standard derivation (RSD) was determined by the hexaplicate analysis of the silymarin from Sigma-Aldrich (SM 1), limits of detection (LOD), and limits of quantification (LOQ) were calculated from the following equations: LOD = 3 × h_n_/m, LOQ = 10 × h_n_/m, where h_n_ is the noise of the baseline and m is the slope of the calibration curve.

### 2.4. Semi-Preparative Chromatography

The LC system used for isolation of 2,3-dehydrosilybin and 2,3-dehydrosilychristin consists of an LC-8A preparative HPLC pump, SPD-20A dual-wavelength UV detector, FRC-10A fraction collector, and CBM-20A bus module controlled by the LC solution 1.24 SP1 software (Shimadzu, Kyoto, Japan). 

**Figure A1:**
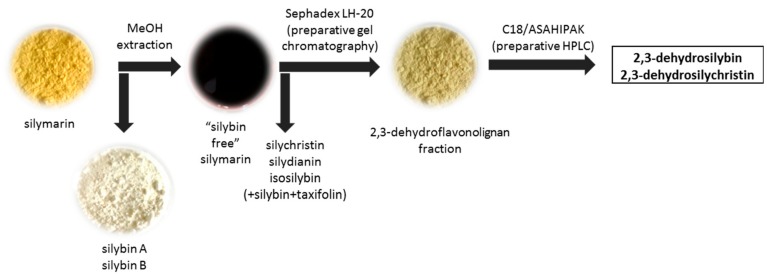
Steps of the sample preparation for isolation, characterization, and quantification of 2,3-dehydrosilybin and 2,3-dehydrosilychristin from silymarin [23].

Semi-preparative chromatography of the fraction enriched in 2,3-dehydroflavonolignans (see Section 2.1 and Figure A1 in Appendix B) was conducted using monolithic Chromolith SemiPrep RP-18e (100 × 10 mm, Merck, Darmstadt, Germany) column with monolithic Chromolith RP-18e (5 × 4.6 mm) pre-column in mobile phases containing either 50% methanol, 0.1% formic acid, 5 mL/min flow rate or 45% methanol, 5% acetonitrile, 0.1% formic acid, 5 mL/min flow rate. Both mobile phases produced similar results. Alternatively, ASAHIPAK GS-10G 7B (300 × 25 mm, 20 μm, Shodex, Munich, Germany) column was used with mobile phase 50% methanol, 0.1% formic acid at 5 mL/min flow rate. The injection volume was 300 µL (37 mg/mL) in all cases.

### 2.5. NMR Spectroscopy

NMR spectroscopy was used to identify 2,3-dehydrosilybin and 2,3-dehydrosilychristin. The spectra were measured on a Bruker AVANCE III 600 and 700 MHz spectrometers in DMSO-*d*_6_ at 30 °C. The signal of solvent was used to reference NMR spectra (*δ*_H_ 2.499 and *δ*_C_ 39.46). The reported assignment (Table A1 in Appendix A) is based on COSY, gHSQC, and gHMBC experiments.

## 3. Results and Discussion

### 3.1. Optimization and Standardization of Analytical Techniques

**Figure 2:**
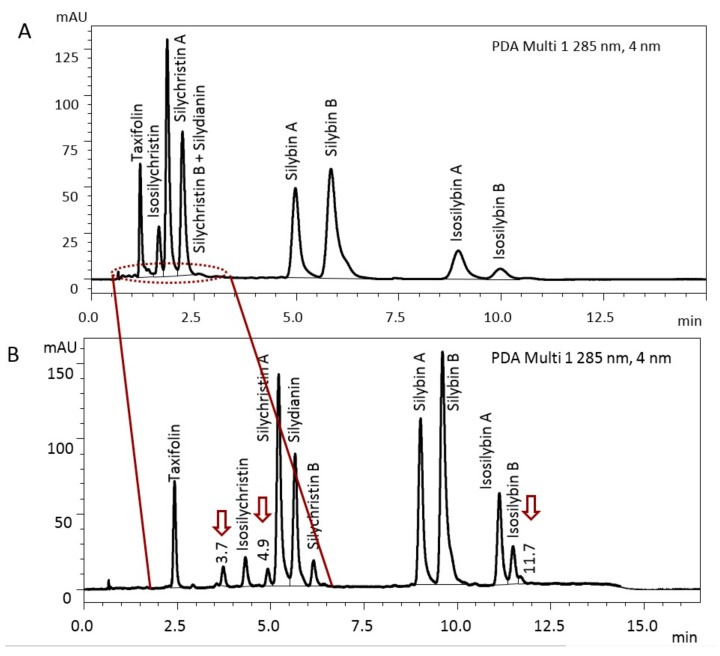
Comparison of the isocratic (**A**) and gradient (**B**) HPLC analytical method for silymarin SM 3. Chromolith RP-18e (100 × 3 mm) column; isocratic conditions: Mobile phase: 2% acetonitrile, 37% methanol, 0.1% formic acid, flow rate 1.1 mL/min, t = 25 °C; gradient conditions: Mobile phase: A = 5% acetonitrile, 0.1% formic acid; B = 80% methanol, 0.1% formic acid; gradient: 0 min 30% B, 12 min 60% B, 13 min 60% B, 14 min 30% B, 16.5 min stop, flow rate 1.1 mL/min, t = 25 °C. The arrows indicate compounds, which have not yet been identified.

Our original isocratic HPLC method for the separation of silymarin components (taxifolin, isosilychristin, silychristin A, silychristin B, silydianin, silybin A, silybin B, isosilybin A and isosilybin B, elution order) was very fast and reliable; however, it allowed only a partial separation of silychristin B and silydianin (Figure 2A). In addition, peaks with higher retention time were blurred due to isocratic elution. 

Therefore, we developed a new quantification method for the individual components of silymarin based on the gradient elution (SM 3 preparation was used to optimize the method). Retention times (R_t_) of taxifolin, isosilychristin, silychristin A, silychristin B, silydianin, silybin A, silybin B, isosilybin A and isosilybin B were 2.4, 4.3, 5.2, 5.7, 6.1, 9.0, 9.6, 11.1, and 11.5 min, respectively (Figure 2B). The identification of taxifolin and flavonolignans was achieved by comparison with the authentic optically pure standards isolated and fully characterized in the laboratory. The improved gradient method sharpened the peaks and mainly allowed the peak distribution in the time scale of 1–2.5 min. In addition to the baseline separation of taxifolin, isosilychristin, silychristin A, silychristin B and silydianin, three other, hitherto hidden substances eluting at 3.7, 4.9, and 11.7 min were separated (marked with arrows in Figure 2B). All these so far unidentified substances have an absorption maximum of 287 nm and are presumably isomeric derivatives of the silymarin flavonolignans. All standards of flavonolignans available in the laboratory were tested to identify these substances and none of them was silyhermin (R_t_ 8.6 min), 2,3-*cis*-silybin A (9.3 min), 10,11-*cis*-silybin B (R_t_ 9.3 min), or 2,3-*cis*-silybin B (R_t_ 10.0 min).

The separation of isosilychristin from silychristin is rare, isosilychristin usually migrates with silychristins A and/or B. The HPLC separation of isosilychristin from silychristin was observed previously [2,26], but separation of silychristin A and silychristin B was not achieved in these works. Similarly, AbouZid et al. separated isosilychristin and silychristin A, but silychristin B was not detected [27]. Kim et al. managed to isolate isosilychristin and silychristin A but not silychristin B using preparative HPLC [28]. Here, we present complete and baseline separation of the individual flavonolignans isosilychristin, silychristin A, and silychristin B.

**Figure 3:**
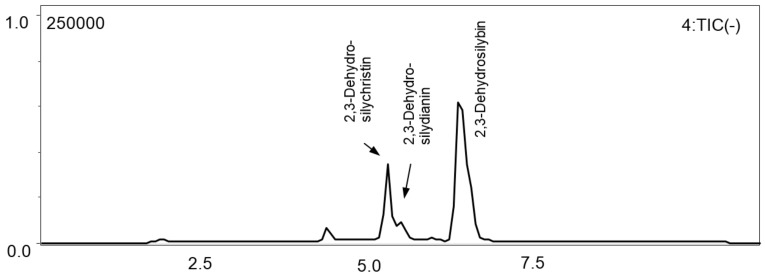
Quantification of 2,3-dehydroflavonolignans by LC-MS with mobile phase A = 5% acetonitrile, 0.1% formic acid, B = 80% acetonitrile, 0.1% formic acid, and gradient: 0 min 20% B, 5 min 90% B, 6 min 90% B, 8–10 min 20% B; flow rate 0.4 mL/min, 25 °C, ESI-MS detection in negative mode at 479.0–479.1 *m*/*z*.

The physical properties (retention time, extinction coefficient, λ_max_ = 255, 375 nm) of 2,3-dehydroflavonolignas differ substantially from those of flavanonols and flavonolignans. These 2,3-dehydroderivatives are minorities in silymarin and are usually not taken into account. Therefore, we have opted for an LC-MS method for their determination and quantification. We have optimized the gradient method to separate 2,3-dehydrosilybin, 2,3-dehydrosilychristin, as well as 2,3-dehydrosilydianin, which was, however, not detected in any of the silymarin samples available in the present study. Retention times of the 2,3-dehydroflavonolignans were 6.4, 5.5, and 5.3 min, respectively (Figure 3). 2,3-Dehydrosilydianin was then used as an internal standard for the quantification of 2,3-dehydrosilybin and 2,3-dehydrosilychristin in silymarin preparations (see Section 3.4.). 

### 3.2. Calibration and Validation of the Method

Seven-point calibration curves of silymarin components were constructed; the slopes, R^2^, LOD, LOQ, and RSD were calculated (Table 1). The results in Table 1 show that the slopes of most of the flavonolignans differ only slightly with the exception of the flavanonol taxifolin (higher) and flavonolignans silydianin and silychristin B (lower). This result, in line with previously published reports [2,26], clearly shows that the calibration curves strictly for each individual silymarin component must be measured to precisely determine their content. Generalization of one calibration curve, such as analysis based only on the most common compound silybin [3,27,29], leads to gross inaccuracies in the quantification.

### 3.3. Separation of Enantiomers of 2,3-Dehydrosilybin and 2,3-Dehydrosilychristin

**Figure 4:**
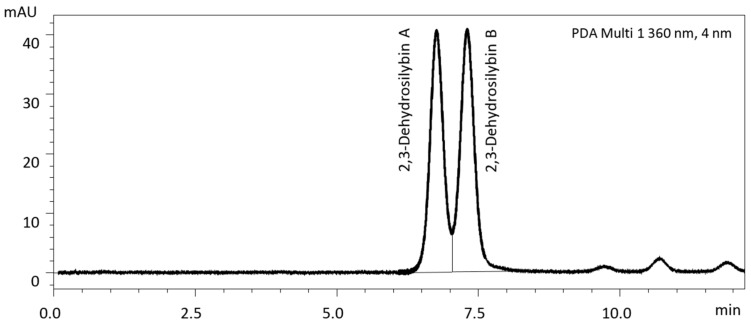
Separation of 2,3-dehydrosilybin enantiomers using chiral column Lux 3μ Cellulose-4 (2 × 50 mm) and pre-column (4 × 2 mm) and binary gradient elution with mobile phase A: 10% acetonitrile, 0.1% formic acid, and B: 80% acetonitrile, 0.1% formic acid; gradient: 0 min 30% B, 0–12 min 30–50% B, 12–13 min 50–30% B, 13–15 min 30% B; flow rate 0.5 mL/min, t = 25 °C.

2,3-Dehydrosilybin and 2,3-dehydrosilychristin naturally occur as enantiomers A and B, which are not separable by the analytical methods used above and, to the best of our knowledge, their separation has not been published yet. Therefore, we developed a new analytical method based on the chiral separation. Quasi-baseline separation of the two enantiomers of 2,3-dehydrosilybin was achieved for the first time; the enantiomers ratio A:B is 48:52 (Figure 4). However, when the same method was applied to the separation of 2,3-dehydrosilychristin (R_t_ 2.7 min) and 2,3-dehydroisosilybin (R_t_ 7.8 min) enantiomers it failed despite numerous attempts of its further optimization.

### 3.4. Flavonoid, Flavonolignans, and Dehydroflavonolignans Quantification

**Figure 5:**
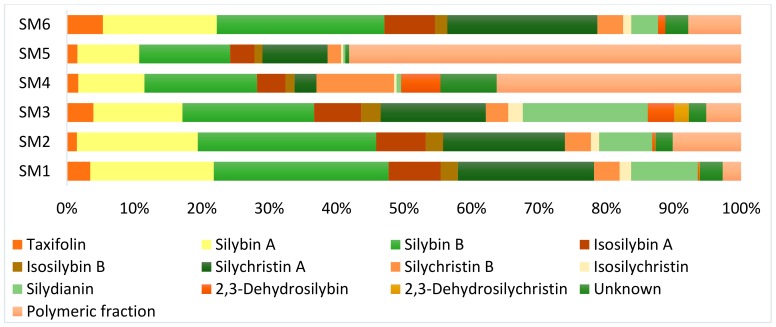
Percentage of individual components in silymarin preparations SM 1–SM 6. Individual silymarin components were quantified using calibration curves of optically pure standards isolated or prepared in house. The content of the polymeric fraction was determined as the mass difference between the content of the individual flavonoid, flavonolignans, and 2,3-dehydroflavonolignans and the mass concentration of the complex extracts.

The content of flavonoid, flavonolignans, and 2,3-dehydroflavonolignans in silymarin preparations SM 1–SM 6 was calculated using the slopes from the calibration curves for each individual compound (Table 1, Figure 5). Coniferyl alcohol (R_t_ 1.6 min) was found in none of the silymarin preparations analyzed. In an agreement with the literature [27,30,31], reviewed in [7] and [18,19], the content of individual substances in silymarin preparations was found to vary considerably (Table 2, Figure 5).

The ratio of regioisomers of silychristin A/B was found to be ca 5:1 in all silymarin preparations except the sample SM 4 where the ratio is nearly reversed (1:3.5). The silybin A and B ratio is very similar in all silymarin samples tested (4:6–3:7), as well as the ratio of isosilybin A/isosilybin B (ratio in range 2.4:1–3.2:1). The content of the flavanonol taxifolin varied from 2.5 mg/g in SM 5 to 31 mg/g in silymarin SM 6. The lowest content of isosilychristin was found in preparations SM 4 and SM 5 (0.9 and 0.5 mg/g, respectively), while the highest was in SM 1 and SM 3 (13 and 15 mg/g, respectively). The highest silydianin content was recorded in SM 3 (131 mg/g), the lowest at SM 5 preparation (0.5 mg/g). The concentration of three unknown compounds in silymarin (marked with arrows in Figure 1) was calculated using the average slope of all available flavonolignan standards because of their identical absorption spectrum (λ_max_ = 287 nm) and is therefore burdened with an error. The three unknown compounds were detected in SM 1, SM 3, and SM 4 preparations at 3.7, 4.9, and 11.7 min, respectively, corresponding to [M-H]^−^ *m*/*z* 481 in all three cases. In SM 2, SM 5, and SM 6 preparations, only peaks at 3.7 and 11.7 min were observed (Table 2).

The content of 2,3-dehydroflavonolignans is usually one order of magnitude lower than that of flavonolignans. Moreover, their physico-chemical properties (and consequently also chromatographic ones) differ from those of flavanonol and flavonolignans in silymarin. Their retention time is ca 10 min when using the optimized gradient method for flavonolignans and they are therefore obscured by the peaks of silybins and isosilybins. As their molecular weight (MW) differ from that of the flavonolignans, their detection and quantification in silymarin preparations is possible with a mass detector using an optimized HPLC method for 2,3-dehydroflavonolignans. 

Quite a high content of 2,3-dehydroflavonolignans was found in the preparation SM 3 (28 mg/g of 2,3-dehydrosilybin and 15 mg/g of 2,3-dehydrosilychristin) and also in SM 4 (14 mg/g of 2,3-dehydrosilybin). No other 2,3-dehydroflavonolignans (e.g., 2,3-dehydroisosilybin or 2,3-dehydrosilydianin) with the identical MW were found in silymarin preparations. As far as we know, 2,3-dehydroflavonolignans in silymarin were previously identified only by HPLC-MS [32], but as a peak containing various isomers without baseline separation or comparison with standards and quantification. In addition to this, only two of our previous very recent works [16,18] reported on the content of these minor components in silymarin preparations: 2,3-Dehydrosilybin (0–5%) was found in 26 silymarin samples using UHPLC-MS on a RP-column with high resolution tandem mass spectrometer, but 2,3-dehydrosilychristin or other 2,3-dehydroflavonolignans were not reported [18]. 2,3-Dehydrosilychristin (0.56%) and 2,3-dehydrosilybin (0.33%) in one silymarin sample [16] were determined by HPLC-MS with a RP-18e column and gradient elution [15].

### 3.5. Polymeric Fraction

The content of the polymeric fraction was calculated as the mass difference between the content of all detectable low-molecular silymarin components and the total mass of the respective preparation. The polymers accounted for 22.0%, 36.6%, and 46% of silymarin preparations SM 1, SM 3, and SM 6, respectively, which is in a good agreement with previous reports in literature [7,33]. In contrast, the (apparent) content of polymers was much higher in the case of SM 2 (53%), SM 4 (85%), and SM 5 (93%, Table 2). A high percentage of the polymeric fraction in SM 4 may be due to the addition of a solubility enhancer (organic amines) for improving solubility in water. In the case of SM 5, which was prepared by Soxhlet extraction of silymarin containing pills in acetone, declared additives such as sucrose and lactose probably partially dissolved in the extraction solvents thus increasing the apparent content of polymers.

### 3.6. Isolation and Identification of 2,3-Dehydroflavonolignans Using Semi-Preparative Chromatography

The silymarin SM 2 fraction containing concentrated 2,3-dehydroflavonolignans was separated using a semi-preparative chromatography in order to isolate and definitely confirm the presence of the individual 2,3-dehydroflavonolignans and their possible derivatives (Figure A1). Semi-preparative C18 and preparative ASAHIPACK columns were used and after optimizing the mobile phases and the conditions, 2,3-dehydrosilybin and 2,3-dehydrosilychristin were isolated in sufficient amounts and purities to confirm their structures using HPLC, MS, and NMR (Table A1).

## 4. Conclusions

A new HPLC and LC-MS analytical method for determination and quantification of silymarin components including 2,3-dehydroflavonolignans was developed and validated. Although flavonolignans in silymarin have the same absorption maximum we demonstrated that their slopes obtained from calibration curves vary substantially. We have clearly demonstrated that every silymarin preparation has quite unique composition and therefore accurate analytical characterization of silymarin components is a fundamental step before evaluation of any biological or biophysical activity. The finding of three hitherto unknown substances could open a new opportunity in the field of drug discovery. Their isolation and characterization will be the subject of our future work. Our novel method can be now used extensively, e.g., in the industry for more detailed and accurate description of every silymarin batch made. This is also the first report on HPLC separation of enantiomers of 2,3-dehydrosilybin A and B.

## Figures and Tables

**Table 1:** Parameters of the calibration curves.

Standard	Purity [%]	Slope [m] *	R^2^	LOD [mg/mL]	LOQ [mg/mL]	RSD [%]
Taxifolin	95.4	2,627,107	0.998	0.0010	0.0032	1.1
Isosilychristin	96.7	1,722,853	0.999	0.0015	0.0049	1.3
Silychristin A	99.0	2,020,320	0.999	0.0012	0.0043	1.3
Silychristin B	88.0	1,218,423	0.999	0.0021	0.0070	1.1
Silydianin	97.0	1,438,790	0.999	0.0025	0.0084	1.2
Silybin A	98.0	2,132,608	0.997	0.0014	0.0046	0.8
Silybin B	100.0	2,464,791	0.999	0.0010	0.0033	0.8
Isosilybin A	96.0	2,152,112	0.996	0.0014	0.0045	0.7
Isosilybin B	98.0	2,282,544	0.994	0.0015	0.0049	0.6
2,3-Dehydrosilybin	98.0	33,994,912	0.964	0.0001	0.0005	0.4
2,3-Dehydrosilychristin	100.0	41,737,232	0.973	0.0001	0.0003	0.1

Data were obtained from HPLC-DAD and for 2,3-dehydroflavonolignans from LC-MS. R^2^: Reliability of regression; LOD: Limit of detection; LOQ: Limit of quantification; RSD: Relative standard deviation; * the equation of the calibration curve linear fit (y = mx + c) was used, c was equal to 0 for all standards.

**Table 2:** The content of individual flavonoid, flavonolignans, and 2,3-dehydroflavonolignans in silymarin preparations.

Component	R_t_	[M-H]^−^	Content [mg/g] ^a^					
	[min]	*m*/*z*	SM 1 ^b^	SM 2 ^c^	SM 3 ^d^	SM 4 ^e^	SM 5 ^f^	SM 6 ^g^
Taxifolin	2.4	303	27.8	7.8	27.9	4.0	2.5	31.4
Isosilychristin	4.3	481	13.6	6.4	15.2	0.9	0.5	7.0
Silychristin A	5.2	481	162.0	94.6	110.4	7.6	15.5	130.8
Silydianin	5.6	481	79.1	41.2	131.3	1.6	0.5	23.4
Silychristin B	6.1	481	30.4	20.1	23.5	27.0	3.2	22.4
Silybin A	9.0	481	146.8	93.8	93.4	23.0	14.7	99.2
Silybin B	9.6	481	207.9	138.4	138.3	39.2	21.6	146.0
Isosilybin A	11.1	481	61.9	38.6	49.4	10.0	5.8	44.0
Isosilybin B	11.4	481	20.5	13.3	20.5	3.1	1.9	10.8
Unknown at 3.7 min	3.7	481	10.9	6.8	7.1	8.8	0.7	16.2
Unknown at 4.9 min	4.9	481	9.7	ND ^h^	6.9	3.3	ND	ND
Unknown at 11.6 min	11.6	481	6.6	6.7	4.3	7.5	0.2	3.8
2,3-Dehydrosilybin	6.4	479	1.7	2.1	27.9	13.7	ND	6.4
2,3-Dehydrosilychristin	5.2	479	0.8	0.4	15.2	ND	ND	ND
Σ flavonoid, flavono-lignans and 2,3-dehy-droflavonolignans			777.1	467.7	628.1	135.7	68.3	542.6
Polymeric fraction (%)			22.0	53.0	36.6	85.1	93.2 ^i^	46.0

Data were obtained from HPLC-DAD and for 2,3-dehydroflavonolignans from LC-MS. ^a^ Six injections were performed for each sample and the standard deviation was in all cases less than 1.5%, ^b^ silymarin from Sigma-Aldrich, ^c^ silymarin from Liaoning Senrong Pharmaceuticals, ^d^ silymarin from Indena, ^e^ silymarin from Panjin Huacheng Pharmaceutical Company with additive for better solubility in water, ^f^ Flavobion, ^g^ silymarin from Panjin Huacheng Pharmaceutical Company, ^h^ not detected, ^i^ high apparent percentage of the polymeric fraction is probably due to the addition of a solubility enhancer. The content of the polymeric fraction (highlighted using pink-gray background in the table) was determined as the mass difference between the content of the individual flavonoid, flavonolignans, and 2,3-dehydroflavonolignans and the mass concentration of the complex extracts.

## Appendix A

**Table A1:** ^13^C and ^1^H NMR data of 2,3-dehydrosilybin and 2,3-dehydrosilychristin.

	2,3-Dehydrosilybin					2,3-Dehydrosilychristin				
Atom Nº	*δ*_C_	m.	*δ*_H_	m.	*J*_HH_ (Hz)	*δ*_C_	m.	*δ*_H_	m.	*J*_HH_ (Hz)
**2**	145.49	s				146.67	s	-		
**3**	n.a.					n.a.		-		
**4**	176.04	s				175.75	s	-		
**4a**	102.87	s				102.88	s	-		
**5**	160.55	s				160.62	s	-		
**6**	98.22	d	6.189	d	1.8	98.12	d	6.187	d	2.0
**7**	164.25	s				163.93	s	-		
**8**	93.46	d	6.450	d	1.8	93.26	d	6.406	d	2.0
**8a**	156.12	s				156.06	s	-		
**10**	78.40	d	4.268	ddd	2.5, 4.6, 7.9	87.40	d	5.544	d	6.6
**11**	75.75	d	4.964	d	7.9	52.88	d	3.553	m	-
**11a**						129.73	s	-		
**12**						115.45	d	7.606	br s	-
**12a**	143.25	s								
**13**	116.01	d	7.786	br s	-	n.a.	s	-		
**14**	123.80	s				115.70	d	7.630	d	1.7
**15**	121.01	d	7.758	br m	-	140.82	s	-		
**15a**						148.60	s	-		
**16**	116.68	d	7.111	d	8.7	132.02	s	-		
**16a**	144.80	s								
**17**	127.14	s				110.44	d	6.960	d	2.0
**18**	111.69	d	7.041	d	2.0	147.51	s	-		
**19**	147.56	s				146.46	s	-		
**20**	147.01	s				115.29	d	6.771	d	8.1
**21**	115.24	d	6.819	d	8.0	118.68	d	6.811	dd	2.0, 8.1
**22**	120.45	d	6.887	dd	2.0, 8.0	62.89	t	3.740	dd	5.5, 10.9
								3.694	dd	6.7, 10.9
**23**	60.00	t	3.571	dd	2.5, 12.3					
			3.376	dd	4.6, 12.3					
**3-OH**			n.a.					n.a.		
**5-OH**			n.a.			-		12.462	br s	-
**7-OH**			n.a.					n.a.		
**18-OMe**						55.60	q	3.756	s	-
**19-OMe**	55.63	q	3.790	s	-					
**20-OH**			n.a.					n.a.		
**22-OH**						-		n.d.		
**23-OH**			n.d.							
**x-OH**						-		9.575	br s	-
								9.021	br s	-

For Atom Nº, see Figure 1; m.: multiplicity; s: singlet, d: doublet, t: triplet, q: quartet, dd: doublet of doublets, ddd: doublet of doublets of doublets, br s: broad singlet, br m: broad multiplet; n.a.: not assigned, n.d.: not detected.
